# Bilateral Anterior Capsulotomy for the Treatment of Refractory Somatic Symptom Disorder: A Case Report

**DOI:** 10.3389/fnint.2021.721833

**Published:** 2022-01-18

**Authors:** Weibin He, Lingmin Shao, Huiling Wang, Huan Huang, Shudi Zhang, Chenhui Li, Chencheng Zhang, Wei Yi

**Affiliations:** ^1^Department of Neurosurgery, Renmin Hospital of Wuhan University, Wuhan, China; ^2^Department of Psychiatry, Renmin Hospital of Wuhan University, Wuhan, China; ^3^Center for Functional Neurosurgery, Ruijin Hospital, Shanghai Jiao Tong University School of Medicine, Shanghai, China; ^4^Shanghai Research Center for Brain Science and Brain-Inspired Intelligence, Shanghai, China

**Keywords:** somatic symptom disorder, anterior capsulotomy, PHQ15, HAMA, HAMD, anxiety, depression

## Abstract

Somatic symptom disorder (SSD) is a form of mental illness that causes one or more distressing somatic symptoms leading to a significant disruption to everyday life, characterized by excessive thoughts, feelings, or behaviors related to these symptoms. While SSD is characterized by significant discomfort in some parts of the body, these symptoms are not related to any known medical condition and therefore it cannot be diagnosed using any medical instrument examination. Currently available treatments for SSD, including drug therapy and psychotherapy (such as cognitive behavioral therapy), usually improve psychiatric symptoms, but the results are often disappointing. Furthermore, SSD is often comorbid with anxiety and depression (75.1 and 65.7%, respectively). Importantly, interventions targeting the anterior limb of the internal capsule (ALIC; e.g., deep brain stimulation and thermal ablation) can effectively treat various mental disorders, such as refractory obsessive-compulsive disorder, depression, and eating disorders, suggesting that it may also be effective for treating the depressive symptoms associated with SSD comorbidity. In this report, a 65-year-old woman diagnosed with SSD accompanied with depression and anxiety underwent bilateral anterior capsulotomy. The patient complained of nausea and vomiting, swelling of the hilum of the liver for 14 years, weakness of the limbs for 13 years, and burning pain in the esophagus for 1 year. Psychiatric and neuropsychological assessments were conducted to record the severity of the patients' symptoms and the progression of postoperative symptoms. The patient's somatization, depression, and anxiety symptoms as well as quality of life improved significantly and steadily; thus, anti-depressive and anti-anxiety medication were stopped. However, the patient developed new somatization symptoms, including dizziness, headache, and sternal pain, 10 months after the operation. Therefore, the patient resumed taking flupentixol and melitracen in order to control the new symptoms. This study shows that bilateral anterior capsulotomy appears to be a complementary treatment for refractory SSD with depressive and anxiety symptoms. Furthermore, postoperative use of anxiolytic and antidepressant medications may be useful for controlling future somatization symptoms.

## Introduction

Somatic symptom disorder (SSD) is a mental disorder that manifests as one or more distressing somatic symptoms that result in a significant disruption to daily life due to excessive thoughts, feelings, or behaviors related to these symptoms (American Psychiatric Association, 2013). Although SSD is characterized by significant discomfort in some parts of the body, the results from medical tests and physical examinations do not explain the person's symptoms. The incidence of somatoform disorder is reported to be 5–7%, with a male to female ratio of 1:10 (Kurlansik and Maffei, 2016). Importantly, physical disorders need to be ruled out before diagnosing SSD, and patients often consult multiple specialists (including a digestive medicine consultation for digestive tract symptoms). Depending on the focus of the somatic symptoms, patients with SSD are often diagnosed with multiple functional syndromes, such as fibromyalgia, by a rheumatologist, and irritable bowel syndrome by another physician, at different points in time. Finding the cause of SSD is often time-consuming, labor intensive, and expensive. Currently, the most common treatments for SSDs are drug therapy (5-hydroxytryptamine reuptake inhibitors, serotonin and epinephrine reuptake inhibitors, tricyclic antidepressants, and atypical antidepressants), psychological therapy (such as cognitive behavior therapy), and physical therapy, such as electroconvulsive shock (Leong et al., 2015; Liu et al., 2019). Due to the lack of understanding of the pathogenesis of SSD, it has no specific treatment, and treatment is often ineffective. Patients who do not respond to treatments or are unable to tolerate the side effects of medication often experience extreme discomfort and are unable to function.

Neurosurgical interventions, including ablative procedures and deep brain stimulation (DBS), have been used for more than 60 years to treat mental disorders. Previous studies have found that these neurosurgical techniques can effectively treat various mental disorders, including refractory obsessive-compulsive disorder (OCD), depression, and anorexia (Andrade et al., 2010; Christmas et al., 2011; Miocinovic et al., 2013; Wu et al., 2013, 2021; Visser-Vandewalle, 2014; Zhan et al., 2014; Subramanian et al., 2017; Zhang et al., 2017; Liu et al., 2018, 2020; Merkl et al., 2018; Yin et al., 2018; Deng et al., 2019; Wang et al., 2020). Importantly, interventions targeting the anterior limb of the internal capsule (ALIC), including ablative procedures and DBS, are known to be effective for treating refractory severe depression and anxiety. ALIC interventions are thought to reduce the symptoms of depression and anxiety by affecting the cortico-striato-thalamo-cortical (CSTC) circuit (Christmas et al., 2011; van Dijk et al., 2013; Velasques et al., 2014; Langguth et al., 2015; Bergfeld et al., 2016; Subramanian et al., 2017; Wu et al., 2021).

The key to treating SSD lies in treating the mental symptoms, especially the symptoms of anxiety and depression. Patients with SSD mainly show discomfort in one or more parts of the body and often have high levels of anxiety regarding their health and somatization symptoms. The incidence rates of comorbid anxiety and depression are also high, at 75.1 and 65.7%, respectively (Bekhuis et al., 2016; De Vroege et al., 2018, 2019). Clinically, the more difficult problem is that comprehensive treatments (such as drug and psychological treatments) often provide no noticeable therapeutic effects. To date, no study has reported a successful neurosurgical treatment for this subset of patients. In this report, one patient with refractory SSD accompanied by depression and anxiety was effectively treated.

## Case Report

A previously healthy 65-year-old woman who presented with multiple somatic symptoms was hospitalized. In 2006, the patient presented with nausea, vomiting, abdominal pain, and diarrhea (no symptoms were found among the other diners). After antidiarrheal and spasmolysis treatment was provided, her diarrhea symptoms improved, but the symptoms of nausea, vomiting, and abdominal pain did not significantly improve, and a sense of anal distension gradually appeared.

She was admitted to the gastroenterology department at another hospital, and all examinations (gastroenteroscopy, abdomen-thorax computed tomography (CT) and magnetic resonance imaging (MRI), and stool routine) were normal. Antidiarrheal, climacteric-relevant medicaments were administered, and the symptoms slightly improved. In 2007, she experienced weakness of limbs, soreness, and difficulty falling asleep (1 h after going to bed, she still could not fall asleep). After treatment with Chinese medicine and massages, the symptoms did not improve, and the patient lost 10 kg. From 2007–2011, the patient repeatedly consulted the Departments of Gastroenterology and Traditional Chinese Medicine. No organic pathological changes were observed on repeated gastrointestinal endoscopy, abdomen-thorax CT, MRI, and various other laboratory tests. She was prescribed numerous medications (detailed medications are not known), but the symptoms did not improve. The patient was unable to live and work normally. Then, in 2011, she was admitted to the psychiatric department with a diagnosis of “SSD with depressive and anxiety symptoms.” She received various medications and felt that her symptoms (abdominal pain, abdominal distension, anal distension, weakness, and distension of the limbs) improved to a greater degree when she took flupentixol and melitracen (one tablet, b.i.d.). From 2011–2018, the patient took flupentixol and melitracen and sleep medication, and her physical symptoms were stably controlled. In August of 2018, the efficacy of the flupentixol and melitracen decreased, and the symptoms worsened. In April of 2019, she was admitted to the psychiatric department of our hospital. She received various medications (duloxetine hydrochloride [40 mg, b.i.d.], lorazepam [1 mg, q.n.], buspirone hydrochloride tablets [10 mg, b.i.d.], agomelatine [50, mg q.n.], dexzopiclone tablets [1.5 mg, q.n.], and flupentixol and melitracen [10 mg, b.i.d.]). After discharge, she was asked to continue taking the medications mentioned above for nearly a year, and she felt that her somatic symptoms (such as esophageal burning pain, abdominal distension, anal falling distension, chest tightness, limb weakness) did not significantly improve and had even increased. Because the patient felt that her quality of life was seriously reduced, she considered suicide.

In April 2020, the patient visited the Department of Neurosurgery at the Renmin Hospital of Wuhan University. A multidisciplinary team consisting of various specialists, including those from the psychiatric, neurosurgery, neurology, gastroenterology, anesthesiology, and medical imaging departments, reviewed the patient's medical history, neuropsychological assessments, and psychiatric diagnoses. Except for low levels of total protein (49.7 g/L, reference range: 65-85 g/L), albumin (32 g/L, reference range: 40-55 g/L), and globulin (17.7 g/L, reference range: 20-40 g/L), no significant abnormalities were observed in the laboratory results. A preoperative cranial MRI and CT examination of the chest and abdomen excluded organic diseases of the brain, chest, and abdomen. Before the onset of symptoms, the patient had no stressful events in life, trauma, or other psychosocial factors. She also had no previous mental illness, no family history of mental illness, and no history of psychotropic drug use. The psychiatric team assessed the patient's cognitive symptoms and cognition. The results were as follows: Patient Health Questionnaire-15 (PHQ-15): 20 points, Hamilton Rating Scale for Depression-17 (HAMD-17): 32 points, Hamilton Anxiety Rating Scale-14 (HAMA-14): 28 points, Symptom Checklist-90 (SCL-90): 156 points, Mini-Mental State Examination (MMSE): 27 points, and Pittsburgh Sleep Quality Index (PSQI): 27 points. The mental status examination conducted before surgery revealed that her thought content was logical. Cognitively, she was alert, able to concentrate, and capable of recalling and exchanging new information during the interview. She was fully able to understand and respond appropriately to the information within the consent form. Based on this, the patient was diagnosed with severe somatic symptoms accompanied by severe symptoms of depression, anxiety, and sleep disorders.

Cognitive-behavioral therapy and electroconvulsive therapy were considered but declined by the patient, and there were therefore no good treatment options left. The neurosurgical team concluded that given how the depression and anxiety symptoms seriously affect her quality of life and since multiple courses of antipsychotic drugs have been ineffective, surgery can be attempted. The surgical procedure, including the risks and benefits associated with the operation, and alternative treatment options were repeatedly explained to the patient and her family. The risks inherent to neurosurgery, including death, intracranial hematoma, infection, epilepsy, cerebrospinal fluid leakage, and anesthetic complications as well as the possible side effects of surgery (urinary incontinence, cognitive or executive dysfunction, and personality changes, such as apathy and lack of energy or increased disinhibition and impulsivity) were also described in detail.

Considering the patient's financial status, and her inability to have frequent, long-term, face-to-face follow-ups as well as the parameter optimization procedures specified in the DBS standardized study protocol, after careful consideration, she and her family opted for bilateral anterior capsulotomy and provided verbal and written informed consent.

**Figure 1:**
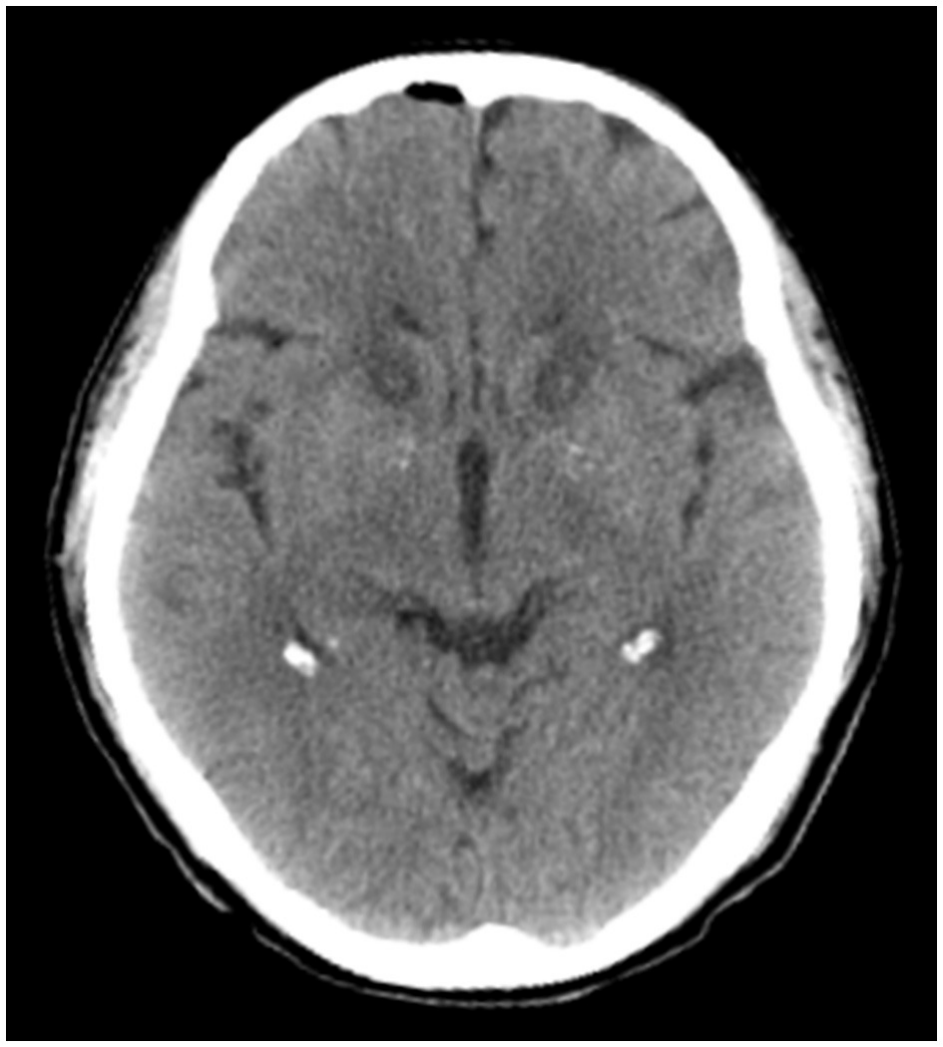
A computed tomography slice of representative ALIC lesions created by bilateral anterior capsulotomy, taken on postoperative day 1.

Before the surgery, the patient underwent a localized MRI (Discovery 750 W Silent, GE Healthcare, Milwaukee, WI, USA) without a head frame. The Leksell stereotactic frame (Elekta Inc., Stockholm, Sweden) was installed on the patient's head under local anesthesia. Immediately after frame installation, the patient underwent a thin-slice CT scan of the head. The located MRI and CT images were imported into the Leksell Surgiplan workstation (Elekta Inc., Stockholm, Sweden) to calculate the target coordinates of the forelimbs of the bilateral internal capsule. Bilateral burr holes were made in front of the coronal suture in accordance with the calculated target coordinates. After the dura mater was opened and cauterized, a 1.6 mm diameter, 4-mm tip non-insulated radiofrequency electrode (Beiqi Medical Technology Co., Ltd., Beijing, China) was placed in the ALIC. A Beiqi RF thermocoagulation instrument (R-2000BA1 model, Beiqi Medical Technology Co., Ltd., Beijing, China) was connected, and the impedance required to complete 60 s of ablative procedures at 75 °C was measured. After cooling to below 40°, the electrode is withdrawn 2-mm and the ablation procedure was repeated 4–5 times to ensure complete ablation of the target. During each lesioning, neurological testing is carried out to rule out impairment of motor or sensory functions. Finally, a lesion 14 mm in length and approximately 4 mm in diameter was formed in each ALIC (Figure 1).

After surgery, to reduce bias in the evaluation, two separate teams of psychiatrists (groups A and B) were formed, such that if group A recommended the patient for surgery, group B evaluated the results after surgery. The evaluation team evaluated the patients' clinical symptoms, social function, and mental state using the MMSE, HAMA, HAMD, SCL-90, World Health Organization Quality of Life-Brief (WHOQOL-BREF), PHQ-15, and PSQI at different follow-up times (1, 6, 10, and 12 months after surgery). In particular, the neurosurgical team used a structured questionnaire consisting of 26 questions to assess surgery-related side effects at the 1-month follow-up.

On postoperative day one, a head CT showed a small amount of intracranial gas, but no intracranial hemorrhage was found (Figure 1). Mild somnolence was observed on day one, but it disappeared on day three. In addition, on postoperative day three, the patient reported a 60% improvement in somatic symptoms. One week after the operation, the patient's vital signs were normal. Since preoperative antipsychotic therapy did not significantly improve the patient's somatization symptoms, and the perceived symptoms were significantly controlled at discharge, the patient was not treated with antianxiety or antidepressant drugs after discharge, only receiving an intermittent sleep-enhancing drug (dexzopiclone, 1.5–3 mg/day).

**Figure 2:**
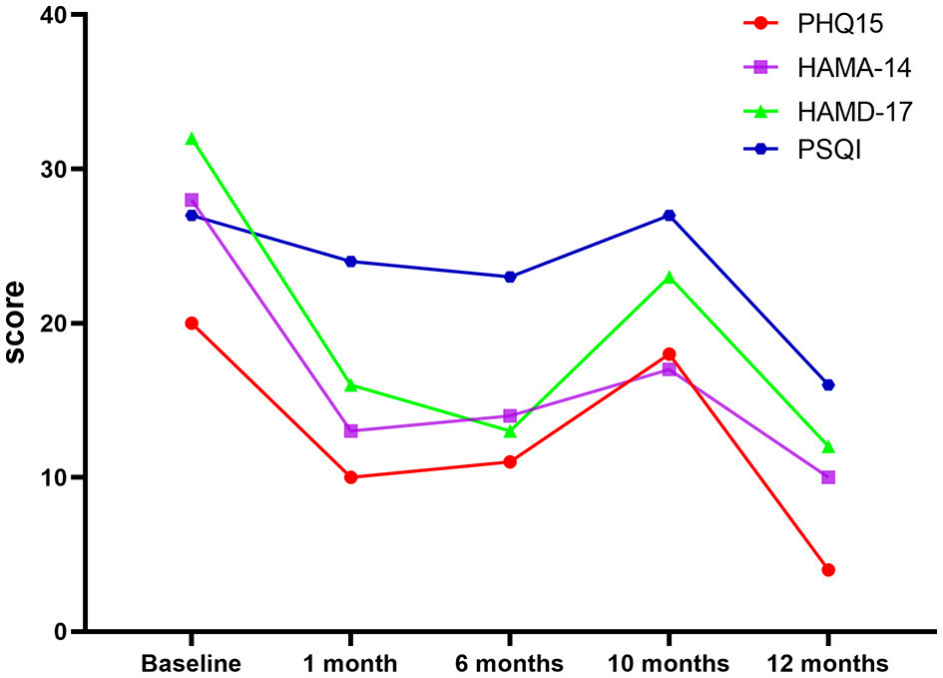
Graph of PHQ15, HAMA-14, HAMD-17, and PSQI scores. PHQ-15, Health Questionnaire-15; HAMD-17, Hamilton Depression Scale-17; HAMA-14, Hamilton Anxiety Scale-14; PSQI, Pittsburgh Sleep Quality Index; WHOQOL-BREF, World Health Organization Quality of Life-Brief; SCL-90, Symptom Check List 90.

The patient was followed up regularly after discharge from the hospital. Table 1 presents the results of the preoperative and postoperative clinical symptom scores. At the 1-month follow-up in the clinic, the neurosurgical team determined no common surgical complications, such as decreased initiative, urinary incontinence, intracranial bleeding, seizures, and incontinence. Although the patient's HAMA, PHQ-15, HAMD, and SCL-90 scores significantly improved, her MMSE score did not decrease. Six months after the operation, the patient was followed up again. No significant change was found in the somatization, depression, and anxiety scores compared with the scores at one month after the operation (Table 1 and Figure 2).

**Table 1:** Assessment of the patient with SSD before and after bilateral capsulotomy.

**Assessment**	**Baseline**	**1 month**	**6 months**	**10 months**	**12 months**
PHQ-15	20	10	11	18	4
HAMD-17	32	16	13	23	12
HAMA-14	28	13	14	17	10
PSQI	27	24	23	27	16
MMSE	27	28	28	27	27
**WHOQOL-BREF**					
Physical field	4.57	11.43	10.29	4.57	17.71
Psychological field	5.33	12.00	10.67	4.67	16.67
Field of social relations	4.00	9.33	9.33	4.00	14.67
Environmental field	11.50	15.00	12.00	12.50	17.50
**SCL-90**					
Somatization	37	20	20	31	14
Obsession	14	12	13	13	12
Interpersonal relationship	10	9	10	11	10
Depression	27	15	16	19	13
Anxiety	16	10	10	14	10
Hostile aggression	6	6	6	6	6
Horrible	7	7	7	7	7
Stubborn	7	6	6	6	6
Psychotic	12	12	10	12	10
Sleep disorders and poor diet	20	13	14	15	13
Total	156	110	112	134	101

*PHQ-15, Health Questionnaire-15; HAMD-17, Hamilton Depression Scale-17; HAMA-14, Hamilton Anxiety Scale-14; PSQI, Pittsburgh Sleep Quality Index; WHOQOL-BREF, World Health Organization Quality of Life-Brief; SCL-90, Symptom Check List 90*.

Ten months after the operation, the patient's gastrointestinal symptoms (nausea, vomiting, anal distension, abdominal pain, abdominal distension, and esophageal burning pain) as well as respiratory and circulatory symptoms (chest tightness) remained under effective control. Unfortunately, the patient developed new symptoms (including dizziness, headache, and sternal pain), while weakness and soreness developed gradually (like those of a cold); however, they were milder than before the surgery. The psychiatric team assessed the symptoms and found that the HAMA, PHQ-15, HAMD, and SCL-90 scores were significantly worse than those observed six months after the operation. The psychiatrists recommended re-prescribing flupentixol and melitracen (10 mg, b.i.d.). Fortunately, the patient reported gradual relief of somatization symptoms after three days. On day ten, she reported experience an almost complete improvement in headache, dizziness, sternal pain, limb weakness, and soreness.

After taking the medicine for two months (one year after the operation), the patient's somatization symptoms were effectively controlled. The severity of the somatic, depression, and anxiety symptoms were significantly reduced (severity of somatic symptoms, very severe before the operation vs. normal at one year after operation; severity of depression, very severe before the operation vs. mild at one year after operation; severity of anxiety, severe before the operation vs. mild at one year after operation; Table 1). The evaluation scores also improved significantly (Table 1 and Figure 2) and were better than those observed in the early postoperative period (1 and 6 months after the operation). Moreover, the patient's quality of life improved significantly.

## Discussion

To the best of our knowledge, this is the first study to report on the surgical treatment of SSD. In this study, the gastrointestinal symptoms (nausea, anal distension, abdominal distension, and burning sensation in the esophagus) and respiratory and circulatory symptoms (chest tightness) rapidly and significantly improved after the operation. Weakness of the limbs were also relieved. Furthermore, the patient's depressive and anxiety symptoms rapidly improved. Previous studies have shown that an anterior capsulotomy can effectively treat various mental disorders, including OCD and depression (Nuttin et al., 2014). However, this is the first patient with SSD to undergo anterior capsulotomy treatment consulted by our multidisciplinary team. The rationale for this was as follows: (1) The patient had been treated with multiple courses of antipsychotic drugs but symptoms had not significantly improved; and (2) in this study, the patient presented with severe depression and anxiety, which severely affected her life before surgery. Therefore, we decided that the best approach for improving the symptoms of depression and anxiety in this patient was through surgery.

The specific mechanism(s) underlying the effect of anterior capsulotomy on somatic symptoms is unclear. First, the etiology and mechanism of SSD remain to be elucidated. Dysfunctions in the CSTC circuit (including sensorimotor loops, cognitive loops, and limbic loops) are possible mechanisms underlying SSD (Ou et al., 2018, 2019). In fact, functional and structural changes in the limbic loops have been found to be associated with non-traumatic somatic symptoms such as pain (Browning et al., 2011; Otti et al., 2013). Second, ALIC is a point of convergence for key white matter fibers connecting the prefrontal and anterior cingulate cortices with the hippocampus, amygdala, and thalamus (Mithani et al., 2020). Previous evidence suggest that ALIC interventions, including ablative procedures and DBS, are effective for various refractory mental disorders, such as OCD, depression, and non-compulsive anxiety (Ruck et al., 2003; Langguth et al., 2015; Mithani et al., 2020). Therefore, anterior capsulotomy was hypothesized to lead to a significant improvement in psychiatric symptoms by affecting the CSTC circuit. However, no previous studies on anterior capsulotomy or DBS in SSD have been reported. Future studies using diffusion tensor imaging and resting-state fMRI are needed to provide more detailed information on the changes in brain circuits that occur in patients with SSD after anterior capsulotomy.

The patient suffered from severe somatization accompanied by severe depression and anxiety. The effects of the surgery on her physical symptoms, depression, and anxiety did not seem to be completely independent. Thus, the rapid improvement in somatization likely led to improved depressive and anxiety symptoms in the patient due to the following reasons: (1) before surgery, the patient had not experienced any relief in somatic symptoms from the various medications prescribed to improve her symptoms of depression and anxiety, and in fact, the patient's symptoms improved steadily when she was not taking antidepressants or anxiety medications after surgery; and (2) As the patient's physical symptoms improved, her depression and anxiety scores also improved. This phenomenon may be better understood by studying whether an anterior capsulotomy also positively affects patients with simple SSD. Interestingly, in 2015, Langguth et al. treated a patient with OCD comorbid with irritable bowel syndrome using ALIC-DBS. The patient's abdominal pain almost completely disappeared after surgery, and their bowel habits normalized to approximately one bowel movement per day (Langguth et al., 2015).

Compared with the surgical side effects (e.g., delirium, disinhibition, and urinary incontinence) previously reported in the literature (Lapidus et al., 2013), the patient's postoperative side effects (only transient drowsiness) were relatively mild. Future studies are needed to determine whether the mild postoperative side effects are related to the use of a relatively small-diameter radiofrequency electrode (1.6 mm).

This study has some limitations. Although the MMSE score showed no impairment of cognitive function, more detailed and longer observation of cognitive function is needed. As no preoperative personality-related assessment was conducted, it was not possible to accurately determine whether the patient's personality changed. In addition, the patient developed new somatic symptoms 10 months after the surgery. Although the new somatic symptoms were brought back under control with medication, their long-term efficacy is worth exploring in a longer follow-up study. Interestingly, the use of flupentixol and melitracen before surgery failed, whereas it effectively controlled the symptoms after surgery. Therefore, the mechanism(s) underlying restoration of the therapeutic effects of the medication following internal capsule injury of the forelimbs need to be determined.

Compared with traditional ablative neurosurgical procedures, DBS has apparent advantages, such as reversibility, adjustability, and the potential for placebo-controlled blind studies. Whether DBS is also a treatment option for SSD is worth further exploration.

## Conclusions

Bilateral anterior capsulotomy may be effective in conjunction with psychoactive medication in SSD patients with psychiatric comorbidities. In this case, the adverse effects were mild and no evidence of neurocognitive impairment was observed.

## Data Availability Statement

The original contributions presented in the study are included in the article/supplementary material, further inquiries can be directed to the corresponding author/s.

## Ethics Statement

The studies involving human participants were reviewed and approved by Clinical Research Ethics Committee of Renmin Hospital of Wuhan University. The patients/participants provided their written informed consent to participate in this study. Written informed consent was obtained from the individual(s) for the publication of any potentially identifiable images or data included in this article.

## Author Contributions

HW, CZ, and WY: study conceptualization, organization, analysis outcome verification, and critical editing and review of manuscript. WH, LS, HH, and SZ: data collection. WH, HH, CZ, and WY: analysis planning. WH, LS, SZ, and CL: performed data analysis. WH: manuscript preparation. All authors contributed to the article and approved the submitted version.

## Conflict of Interest

The authors declare that the research was conducted in the absence of any commercial or financial relationships that could be construed as a potential conflict of interest.

## Publisher's Note

All claims expressed in this article are solely those of the authors and do not necessarily represent those of their affiliated organizations, or those of the publisher, the editors and the reviewers. Any product that may be evaluated in this article, or claim that may be made by its manufacturer, is not guaranteed or endorsed by the publisher.
